# Implementing a Telehealth Perinatal Depression Prevention Program: Perspectives of Rural and Urban Clinical Staff

**DOI:** 10.1155/jonm/4109971

**Published:** 2026-10-09

**Authors:** Tumilara Aderibigbe, Wen Tu, Ryoko Kausler, Uma Parameswaran Dorn, Sara Simonsen, Nemeh Manasrah, Lauren Gimbel, Marcela Smid, Marcia Williams, Gwen Latendresse

**Affiliations:** ^1^ Department of Nursing University of Massachusetts Boston 100 Morrissey Boulevard Boston Massachusetts USA umb.edu; ^2^ College of Nursing University of Utah Salt Lake City Utah USA utah.edu; ^3^ School of Nursing Boise State University 1910 West University Drive Boise Idaho USA boisestate.edu; ^4^ Department of Psychiatry and Behavioral Sciences Stanford University Stanford California USA stanford.edu; ^5^ Department of Obstetrics and Gynecology University of Utah Health 50 North Medical Drive Salt Lake City Utah USA; ^6^ School of Nursing Cedarville University 251 North Main Street Cedarville Ohio USA cedarville.edu

## Abstract

**Background:**

Nurses are key stakeholders in telehealth intervention implementation. Nurses are also instrumental in the management of perinatal depression, a common complication during pregnancy and postpartum. Telehealth interventions are a promising approach to addressing perinatal depression; however, its successful integration into routine maternal care remains challenging, particularly across diverse clinical settings. This qualitative study examined how registered nurses in the United States and other clinical staff perceived and navigated the implementation of a telehealth perinatal depression prevention program.

**Methods:**

A qualitative descriptive study was conducted with a purposive sample of 24 participants including nurses, medical assistants, lactation consultants, community health workers, and hospital administrators at rural and urban health facilities. Seven virtual focus groups were conducted with participants between March and December 2024. To foreground workflow integration, we focused on nonprovider clinical staff directly involved in patient flow. Focus groups were video‐ and audio‐recorded, transcribed, and analyzed in NVivo (Version 14) using codebook thematic analysis, guided by the Theoretical Domains Framework. Rigor was established and maintained throughout the study.

**Results:**

Facilitators included provider endorsement, training, workflow alignment, and perceived patient benefit. Barriers included stigma, limited technology access and literacy, competing workflow demands, and inconsistent provider engagement. Rural clinical staff underscored privacy concerns and structural digital access barriers, whereas urban staff emphasized provider endorsement and electronic health record integration as central implementation levers. Recommendations focused on strengthening endorsement, improving education and workflow integration, enhancing marketing strategies, and increasing flexibility in intervention delivery.

**Conclusions:**

Addressing barriers is critical for successful integration of telehealth perinatal depression prevention into routine maternal care. Context‐sensitive implementation strategies may mitigate rural–urban inequities in access to digital maternal mental health support.

**Implications for Nursing Management:**

Nurse managers should address workflow constraints and evaluate duty allocation to facilitate smooth incorporation of the telehealth perinatal depression prevention program into routine maternal care.

## 1. Introduction

Perinatal depression (PD) is a prevalent complication during the perinatal period (from pregnancy up to 1 year postpartum [1]). PD affects approximately one in four women globally [2] and is associated with adverse outcomes, including low birthweight and preterm birth among infants [3], and low quality of life and increased suicidal risk among women [3, 4]. These impacts are pronounced in rural areas and among underserved populations, where access to mental health services remains limited [5]. Traditional in‐person mental health services face numerous barriers to access and utilization, including geographic isolation, mental health provider shortage, and persistent stigma surrounding mental health treatment [6, 7].

In response to these challenges, telehealth has emerged as a promising solution for delivering perinatal mental healthcare [8], particularly for women living in rural or frontier areas, where digital delivery may reduce travel burden and expand scheduling flexibility. However, expanding availability does not guarantee integration into routine care. Successful implementation requires alignment of clinical flow, staff engagement, and system‐level support.

The perspectives of key stakeholders—both healthcare providers and patients—are crucial in understanding the potential implementation of telehealth interventions. Healthcare providers, particularly those in public health departments and rural settings, have increasingly supported telehealth as a viable option for perinatal care delivery, noting improvements in quality of care, access to services, and psychosocial support [9]. From the patient perspective, research indicates high acceptability of these interventions, showing that web‐based and mobile health platforms offer psychological resources with advantages such as lower costs, broader reach, and reduced stigma [10, 11]. Among specific populations, including low‐income women, telehealth services have demonstrated both feasibility and high satisfaction levels [12, 13]. Despite these promising findings, significant gaps remain regarding how telehealth services are embedded and implemented across diverse rural and urban healthcare settings.

Information is lacking about the perspectives of nonprovider clinical staff, including medical assistants and nurses, who operationalize referrals, navigate patient flow, and integrate digital tools into everyday practice. Thus, the aim of this study was to examine the perspective of nurses and medical assistants about the implementation of our telehealth PD prevention program in a large urban academic health system and six rural public health district clinics in one Intermountain West state in the United States.

## 2. Methods

### 2.1. Design and Theoretical Framework

This study was part of a larger implementation research project, the Maternal Mental Health Access (MaMa) study [14]. Our team developed the Telehealth Perinatal Depression Prevention Program (TelePDPP), a multicomponent intervention found to reduce perinatal depressive symptoms among pregnant and postpartum women with mild to moderate symptoms of depression [14]. In the present study, TelePDPP was implemented through an online patient education portal (OPEP). The commercially available platform Bump2Baby360 (formerly YoMingo) was the OPEP used to implement the intervention [15]. The TelePDPP contained PD screening questions as well as educational content about PD and also offered Facebook discussion boards and participation in synchronous videoconference support sessions, combining asynchronous education with interactive peer and facilitator support. The present study used a qualitative descriptive design and was informed by the Theoretical Domains Framework (TDF), a framework for identifying behavioral determinants of intervention implementation [16]. The 14 domains of the TDF include knowledge; skills; social/professional role and identity; beliefs about capabilities; optimism; beliefs about consequences; reinforcement; intentions; goals; memory, attention, and decision processes; environmental context and resources; social influences; emotion; and behavioral regulation [16]. The study was conducted and reported according to the Standards for Reporting Qualitative Research (SRQR) [17] (Supporting 1).

### 2.2. Recruitment and Sample

The TelePDPP was implemented in 7 clinical sites. These include a large urban academic health system and six rural public health district clinics. Active and passive recruitment approaches were used. Flyers and newsletters with a survey link, QR code, session time options, and contact information were distributed to clinical staff. Additionally, study clinical coordinators visited clinics in person to encourage interested staff members to join the study. Interested individuals completed an initial interest form via Qualtrics and were invited to participate in a scheduled focus group. A purposive sample of 24 clinical staff with variation in profession and exposure to the TelePDPP participated in focus groups over two recruitment waves during the implementation project. The first wave took place approximately 1 year after the study began, at which point implementation was already underway, meaning that all participants had the opportunity to observe the program. The second wave occurred approximately 6 months after the first. All eligible participants were invited to attend focus groups at both time points. We included individuals who were (1) > 18 years, (2) English‐speaking clinical staff coordinators (e.g., medical assistants and nurses) who work in the University of Utah Health Clinics or any of the collaborating six rural health district clinics, and (3) had access to the internet and Zoom. Input from clinicians including physicians and advanced practice clinicians (nurse midwives, nurse practitioners, physician assistants) was solicited via survey and will be reported elsewhere and therefore was excluded from this report.

### 2.3. Setting

Eligible participants provided informed consent electronically via Research Electronic Data Capture (REDCap). Focus groups were conducted online using Zoom; all participants were in the United States.

### 2.4. Data Collection

Seven online focus groups, lasting 45–60 min, were conducted (3 rural and 4 urban groups) between March and December 2024. Each focus group consisted of at least three participants. Recruitment continued until thematic saturation was achieved [18]. Four authors (Tumilara Aderibigbe, Uma Parameswaran Dorn, Ryoko Kausler, and Wen Tu) facilitated the focus groups using a semistructured focus group guide exploring facilitators, barriers, and recommendations for engaging with the TelePDPP (Supporting 2). Uma Parameswaran Dorn introduced the research team to foster rapport and encourage open dialog. Focus groups were audio‐ and video‐recorded. A trained note‐taker attended sessions to capture observation notes. Each participant received a $50 gift card for their time.

### 2.5. Data Analysis

Focus groups were transcribed verbatim and analyzed by TA, WT, RK, NM, UD, and MW. A codebook was generated after line‐by‐line open coding was completed in Microsoft Word and Dedoose. Each transcript was coded multiple times by the coders and compared. The team resolved disagreements through consensus. Multiple coders, all with experience with telehealth interventions in maternal mental health, contributed to analysis to enhance analytic rigor and minimize individual bias. The codebook was created and stored in Microsoft Excel, and subsequent generation of themes and subthemes was done in NVivo (Version 14) to support organization and theme development. Braun and Clarke’s codebook thematic analysis was used to generate themes inductively while remaining informed by TDF [19].

### 2.6. Ethical Approval

Ethical approval was granted by the Institutional Review Board at the University of Utah (IRB# 00159109). Participants provided informed consent electronically. Data were de‐identified and stored in the University’s secure cloud storage to maintain confidentiality and data security.

### 2.7. Rigor

All authors except Nemeh Manasrah have conducted qualitative research previously on PD. Researchers acknowledged and bracketed prior assumptions during data collection and analysis and maintained reflexivity throughout the study. Researchers have no previous relationship with staff members who participated in the study. All coders have clinical experience; they identified how their clinical experiences may have shaped their interpretation of findings from the study. The study’s trustworthiness was evaluated through its credibility, dependability, confirmability, and transferability. Data credibility was supported through digital recording, team‐based coding, and team review of generated themes. An audit trail of transcripts and analytic memos was maintained to ensure dependability. Reflexive memos about data interpretations and triangulation of analytic perspective enhanced confirmability of findings. Transferability of findings was supported by detailed contextual descriptions and representative quotes. AI was not used in this study.

## 3. Results

### 3.1. Demographic Characteristics of Participants

A total of 24 clinic staff participated in the study. All participants were female. Half were from rural clinics. Most participants were medical assistants (29.2%) or registered nurses (25.0%). More than half had worked in their current roles for over 3 years (54.2%) (Table 1).

**TABLE 1:** Demographic characteristics of participants (*n* = 24).

Variables	Category	Frequency (*N*)	Percentage (%)
Location	Rural	12	50.0
	Urban	5	20.8
	Not reported	7	29.2

Healthcare profession	Medical assistant	7	29.2
	Registered nurse	6	25.0
	Lactation consultant	1	4.1
	Community health technician	1	4.1
	Community health worker	1	4.1
	Grant program manager	1	4.1
	Management services: director	1	4.1
	Not reported	6	25.0

Years of working in current role	< 1 year	2	8.3
	1‐2 years	3	12.5
	3–5 years	7	29.2
	> 6 years	6	25.0
	Not reported	6	25.0

### 3.2. Themes

Consistent with the goal of our study to promote the implementation of our TelePDPP, we were focused on identifying facilitators, barriers, and recommendations. This is why we followed the TDF, which served as a template for our codebook thematic analysis. Three overarching themes were identified from the analysis—Perceived enablers of two‐way utilization, Perceived challenges with two‐way utilization, and Suggestions to promote two‐way utilization. “Perceived enablers of two‐way utilization” and “Perceived challenges with two‐way utilization” each had two subthemes, and “Suggestions to promote two‐way utilization” had five subthemes (Figure 1). Themes were also mapped onto TDF (Table 2). Additional quotes from participants are included in supporting 3.

**FIGURE 1:**
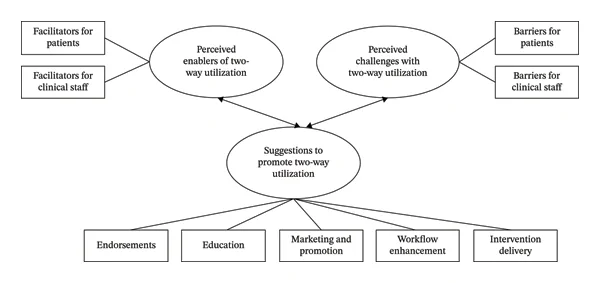
Thematic map of clinical staff–identified barriers, facilitators, and recommendations regarding the implementation of the PD prevention program.

**TABLE 2:** Mapping of the findings onto the theoretical domain framework.

Themes	Subthemes	TBF domains	Examples of quotations
Perceived enablers of two‐way utilization	Facilitators for Patients	‐Optimism	*“… these women want to know where their children are. They want to be good moms. They want the resources…” [Rural staff, 6/20/24]*
		‐Intentions	
	Facilitators for Clinical Staff	‐Environmental context and resources	*“… our clinical services department, they’ve really been the ones spearheading this… when moms or pregnant moms come in for our car seat education and free car seat program, we’re able to have things in our education room, and our education bag. So, we’ve put the [OPEP] information in there around postpartum depression…” [Rural staff, 12/11/24]*
		‐Social/professional role and identity	*“… my experience with the YoMingo app has been introducing patients to it and kind of helping them get it set up and started and then I used it myself during my most recent pregnancy a little bit.” [Urban staff, 10/23/24]*
		‐Intentions	*“…during WIC we give the screening on an iPad to all prenatal and postpartum patients… but then you get to talk about the resources and TelePDPP Study, and then through our Parents as Teachers home visiting program, we started doing the screenings on the iPads so that you guys just get the information right off the bat, and that’s been good too.” [Rural staff, 12/11/24]*
Perceived challenges with two‐way utilization	Barriers for Patients	‐Skills	*“… I agree with A that things are a little bit hard to navigate. I’ve heard that from some patients, that it just can be a little bit difficult to find things…” [Urban staff, 3/13/24]*
		‐Knowledge	
		‐Beliefs about capabilities	
		‐Environmental context and resources	*“… they’re so overwhelmed, there’s so much going on, yeah… the chaos usually of their lives taking over and forgetting to take care of themselves, which we all do. Any mom, any parent.” [Rural staff, 12/11/24]*
		‐Social influences	
		‐Emotion	
	Barriers for Clinical Staff	‐Knowledge	*“I think a barrier that I have like seen in the past for me to even just bring up mental health is a partner who seems standoffish, you know? Partners who are just like on their phone in the corner or just not even paying attention. That really turns me off…” [Urban staff, 10/23/24]*
		‐Skills	
		‐Environmental context and resources	
		‐Social influences	

Suggestions to promote two‐way utilization	Endorsements	‐Reinforcement	*“It’s a stronger message from the doctor and the child’s provider than us and so I think maybe that’s what you do in the rural community, is we’ve got to get to the providers and make sure they’re aware of it and that they have the resources and they’re promoting it and take the time to do so, which they have more time. They have the time and the luxury to spend more one on one with their clients, I think.” [Rural staff, 6/20/24]*
		*-*Social/professional role and identity	
		‐Social influences	
	Education	‐Knowledge	–
		‐Skills	
		‐Social influences	
	Marketing and Promotion	‐Knowledge	*“I was just wondering, is there, at the end of the study, going to be some sort of results we can see of like, “Oh, this many people said that it was helpful” or “It decreased depression in this many people,” that maybe we could help use to publicize it? Say like, “Oh, this is how beneficial other people found it during the study,” so it kind of makes it look good for other people to join the program in the future.” [Urban staff, 3/13/24]*
		‐Social influences	
	Workflow Enhancement	‐Knowledge	*“We create like a newbie packet, we call it, and we can attach that to that… just having it available when they get home and go over everything, they might have more interest in it because it’s just so much to take at the first visit.” [Urban staff, 3/13/24]*
		‐Skills	
		‐Reinforcement	*“… is it possible to like follow up with them, like following up with the client?” [Rural staff, 12/10/24]*
		‐Environmental context and resources	
		‐Social/professional role and identity	
	Intervention Delivery	‐Skills	*“I mean if there was like some type of incentive for the patient, maybe it’s monetary, maybe it’s like even just like you get a cool sticker, like a stupid motivation to like enroll in [the OPEP]. Like you’ll get a star in your chart and your doctor is going to see that might be enough to motivate someone to like actually enroll before they need it… [Urban staff, 10/25/24]*
		‐Reinforcement	
		‐Social influence	
		‐Environmental context and resources	

#### 3.2.1. Perceived Enablers of Two‐Way Utilization

Clinicians reported the factors they perceived were catalysts for patients to learn about PD prevention using the TelePDPP. They also identified factors that motivated them to refer patients to access the TelePDPP for this purpose. Hence, this theme was divided into two subthemes—Facilitators for Patients and Facilitators for Clinical Staff.

##### 3.2.1.1. Facilitators for Patients as Reported by Clinical Staff

Two facilitators (ease of access and desire to educate) were identified for rural patients, and one (endorsement by medical provider) was identified for urban patients. Rural clinical staff perceived that ease of accessing the TelePDPP addresses patients’ concerns about privacy and stigma and expressed that this increased patient engagement:

*“… having the ability to do it from home and join the Facebook group and things like that, it’s a little bit of a better comfort level for our clients in our rural and some of our areas are frontier…” (Rural staff*, *12/10/24)*

For urban patients, endorsement of the OPEP by clinicians increased engagement:

*“… I loved that Dr. [C] always handed out little, I think it was a card… it was something tangible. It was, “Here. Here you go. This is how you do this.” And that was really helpful…” (Urban staff, 10/25/24)*

##### 3.2.1.2. Facilitators for Clinical Staff

Five facilitators were identified for clinical staff: endorsements, personal/professional experience, training, workflow, and app interface. The most common facilitator was endorsement, which could be personal, from patients, or from colleagues.

One clinical staff personally endorsed the OPEP:

*“… I really like it. I think it’s well done, the different areas within the app. So, we want to get this going…” (Rural staff, 6/20/24)*

Others described patient feedback as reinforcing referral behavior:

*“… one of my families did the whole program, and she said it really helped her because she didn’t want to go on medication…” (Rural staff, 12/11/24)*

Colleague enthusiasm also shaped engagement:

*“… my new girl I trained the first time was probably like, “How long has this [OPEP] been here?” Because she was like, “This is so cool. Let’s click on this…” (Urban staff, 10/25/24)*

Beyond endorsement, personal investment in maternal mental health facilitated referrals:

*“… I always emphasize mental health. Mental health, postpartum mental health is super important to me and so I always like to put a plug in there.” (Urban staff, 10/23/24)*

Improved organization within the OPEP enhanced usability and referral confidence:

*“I think the first time I logged in; it was when it was brand new. So, no offense… I clicked around in it… but I feel like it was all over the place. Now I feel like there’s specific categories that tells you where to go and what to do and I think, I think that really helps…”* (*Urban staff, 10/25/24*)

Rural clinical staff emphasized the importance of structured training:

*“I think we′ve had really good training on how to access (OPEP) and the MaMa Study. Which I think has helped keep our staff feel more comfortable with talking about it and referring and accessing…that′s helped us to be more successful at putting it out there.” (Rural staff, 12/11/24)*

Workflow alignment also emerged as critical. Urban staff noted that integration was most feasible when embedded within existing visit structures (e.g., nonstress test appointments, Special Supplemental Nutrition Program for Women, Infants, and Children (WIC) screenings, interpreter‐supported visits):

*“… for us doing the NST appointments, like (A) said, we have the patient in there for at least 20 min, and we do a lot of chatting in that time. So, it’d be easy to throw in something about (OPEP) with every patient.” (Urban staff, 10/23/24)*

Collectively, facilitators reflected the importance of normalization, workflow compatibility, and perceived patient benefit. While rural staff emphasized privacy and access, urban staff highlighted provider endorsement and integration within established clinical systems.

#### 3.2.2. Perceived Challenges With Two‐Way Utilization

Barriers were identified at both patient and staff levels, often reflecting structural, cultural, and workflow‐related constraints.

##### 3.2.2.1. Barriers for Patients as Reported by Clinical Staff

Both rural and urban patients were reported to experience similar barriers including language barriers, limited smartphone access, low awareness about the OPEP, and stigma. However, language barrier and stigma were pronounced among rural patients.

*“The only barrier I can think of sometimes is just having a language barrier… we have a lot of Spanish population… sometimes like, yeah you′re trying to explain it, but sometimes you get blank stares back…” (Rural staff, 12/11/24)*

Limited access to smartphones and low technology literacy also limited engagement:

*“I think a barrier in my (W) clinic is not everybody has a smartphone out there. So, they don′t have the technology to access it. A lot of them don′t… definitely, technology or not having the access to it can be a barrier for our population out there.” (Rural staff, 12/11/24)*

Low awareness about the OPEP further limited uptake:

*“… I would tell a patient about (OPEP) and they’re like, “Yo what? Yo what? I don’t know what you’re talking about” …enrollment, I think, that has gone up dramatically, but I still would say for my population it’s maybe like a 50/50. Or like maybe they’ve downloaded it but not utilized it…” (Urban staff, 10/25/24)*

Stigma was pronounced among rural patients:

*“I think a barrier, especially for my population in (W) could just be admitting and then being okay with saying I have these needs, instead of just shoving it down. Being like, no, culturally you don′t express those feelings, you don′t talk about it. You′re just like, you have to be okay. I mean that can be blanket anybody, in any culture. Not just my Latino or Latinx culture out there…” (Rural staff, 12/11/24)*

Unique to urban patients, urban staff described cultural expectation discouraging outward expressions of emotional vulnerability, as contributing to patients’ reluctance to discuss mental health concerns:

*“…the other day about one of our patients who was obviously having a hard time and her mother was the one, like “Just get to work. Why are you sad? Why are you doing this? You need to just take care of the baby. You need to take care of your six children”…” (Urban staff, 10/23/24)*

##### 3.2.2.2. Barriers for Staff

Three primary staff‐level barriers were identified: workflow, knowledge/experience gap, and perceived app burden.

Workflow and time constraints were the most salient:

*“…It’s so hard because everyone is working like at 150%. So, I don’t know who would take ownness of that…” (Urban staff, 10/25/24)*

Lack of clinician engagement further constrained referrals:

*“I will say the provider that I worked for was not a huge fan of [OPEP], so he was not pushing it. So, we didn’t have a ton of feedback…” (Urban staff, 10/25/24)*

Some rural staff reported limited knowledge and experience with the OPEP:

*“I’ve never used it. I’ve heard of it, but I’ve never used it.” (Rural staff, 10/25/24)*

Finally, staff expressed concern that downloading an additional app may be burdensome for patients, deterring referrals:

*“I think them downloading one extra app is an issue. I mean everything is an app, right?… I think it just makes it a little bit trickier…” (Urban staff, 10/25/24)*

In contrast to facilitators, barriers were more frequently described as structural and systemic rather than individual. Rural staff emphasized privacy, stigma, and digital access limitations, whereas urban staff highlighted provider disengagement and workflow overload as central impediments.

#### 3.2.3. Suggestions to Promote Two‐Way Utilization

This theme was grouped into five subthemes: Endorsement, Education, Marketing and Promotion, Workflow Enhancement, and Intervention Delivery. Collectively, recommendations centered on strengthening endorsement, embedding the TelePDPP within existing clinical workflows, increasing awareness through both in‐person and digital channels, and enhancing flexibility in intervention delivery.

##### 3.2.3.1. Endorsement

Clinical staff consistently emphasized that clinician endorsement was paramount, and could strengthen participants’ engagement with the TelePDPP:

*“…I think it’s just having the providers talk to them about it. Us as MAs can say all that we want to say, but if the provider says it, it’s golden, right…” (Urban staff, 10/25/24)*

For rural patients, endorsement from trusted community members was described as equally influential:

*“… that’s one thing about small communities, is you know a lot of people, so word of mouth goes pretty far. Yep, the connections and the networks.” (Rural staff, 6/20/24)*

Thus, endorsement operated at both institutional and community levels, particularly in smaller rural networks where interpersonal trust shapes care‐seeking behavior.

##### 3.2.3.2. Education

Two forms of education were highlighted for rural patients. First, educating patients about accessing and navigating the OPEP was mentioned:

*“… maybe there already is but is there a “How to access steps to access the screener?” To make it more user friendly I guess.” (Rual staff, 12/11/24)*

Second, addressing myths and misconceptions about PD and privacy concerns were discussed:

*“… there’s definitely a different mindset as far as people knowing your business and feeling like people know what’s going on, even though they probably don’t. It’s just a whole different mindset when you’re in rural and frontier areas… So, I just think if there’s a way to focus on the rural area and that concern that we have for some reason.” (Rural staff, 12/10/24)*

Educational recommendations therefore extended beyond technical instruction to cultural normalization of mental health discussions.

##### 3.2.3.3. Marketing and Promotion

Complementing in‐person approaches, both rural and urban clinical staff recommended leveraging online platforms including social media, Epic Electronic Health Records MyChart Patient Messaging System:

*“Is there like a dot phrase or anything that we can send to them in MyChart for the PD Prevention?” (Urban staff, 3/13/24)*

Rural clinical staff emphasized awareness about the OPEP and the TelePDPP via direct, in‐person measures is a promising strategy:

*“I think it’s raising awareness, absolutely, and helping me kind of spread that with the clinics or let them be aware of how can we give not just the screenings but let’s give them the resources and I think having both, (the TelePDPP and OPEP), is a really good tool, good step in that direction.” (Rural staff, 6/20/24)*

Rural clinical staff further underscored tailoring marketing and promotion information to address concerns about privacy:

*“Is there a way to do a flyer that maybe addresses the anonymity of it?… maybe something that says, you know, “We offer services from the privacy of your home.” (Rural staff, 12/10/24)*

These suggestions reflect a dual strategy: Normalize telehealth through routine clinical channels while simultaneously reinforcing confidentiality in communities where visibility may deter engagement.

##### 3.2.3.4. Workflow Enhancement

Specific cues to action to enhance staff workflow were provided by both rural and urban staff to increase patient engagement with the TelePDPP. First is having trained medical providers such as nurses introduce the TelePDPP:

“…*I feel like there is that background in training medically and conversation wise with our nurses that they′re more prepared to have that deeper conversation…*” *(Rural staff, 12/11/2024)*

Reviewing specific sections in the OPEP may also prevent patients from being overwhelmed:

*“I tell them to make sure when they’re telling patients, oh, they’re concerned about mental health. Tell them to just focus on that mental health wellness piece…” (Urban staff, 10/25/24)*

Third, emphasizing the use of OPEP during staff meetings, incentivizing team members who use the platform, and following up with providers to refer patients to the OPEP:

*““… Hey, who is using YoMingo?” Just like reminder because it is mentioned at several meetings that I go to… like check with your providers and see how they’re utilizing this…” (Urban staff, 10/25/24)*

Both rural and urban staff suggested downloading the OPEP for patients before discharge and sending routine reminders may be helpful:

*“I really do like the idea of just downloading the application when you′re with them to help that and take one less thing away from their to-do list.” (Rural staff, 12/11/24)*

Collectively, these workflow recommendations highlight the need to shift the TelePDPP from an optional add‐on to an integrated component of routine maternal care.

##### 3.2.3.5. Intervention Delivery

Suggestions regarding the mode of delivery of the TelePDPP were provided by urban clinical staff only. Accessing the TelePDPP individually or within a closed, private, Facebook group may offer some level of reassurance for patients:

*“I guess if the patient doesn’t really like to be in a group setting, is there an option to be alone and do this?” (Urban staff, 3/13/24)*

Another clinical staff added that it may be helpful to allow for some flexibility in the level of engagements:

*“… from other groups that I’ve led, I think we allow space for folks to participate to the extent that they’re comfortable. So, if people need to chat, we’ve kind of given them options to kind of, so I’m imagining it’s very similar in that way.” (Urban staff, 3/13/24)*

Finally, introducing artificial intelligence chatbot to facilitate navigation and resource linkage was proposed to enhance accessibility:

*“A text bot where… they literally could just like inquiry ask the question to this AI bot and then it would pop up, like, “Here’s a resource for you. If you’re still feeling like you need to talk to someone, here’s a phone number to call, right.” Like an AI bot…” (Urban staff, 10/25/24)*

These delivery‐focused recommendations reflect an emerging expectation that digital mental health tools be adaptive, responsive, and minimally burdensome to users.

## 4. Discussion

The aim of this study was to examine the perceptions of rural and urban clinical staff about implementing a TelePDPP. Perceived enablers of two‐way utilization (facilitators), perceived challenges with two‐way utilization (barriers), and suggestions to promote two‐way utilization (recommendations) of the TelePDPP, which correspond to 10 domains in the TDF were identified. Four domains of the TDF not captured in the study include beliefs about consequences; memory, attention, and decision process; goal; and behavioral regulation. These domains are not relevant to the context of the present study as they target the ability to remember and follow instructions to achieve a goal and perceived consequences of performing a behavior.

Similar themes were reported in our previous study on women’s experiences with an 8‐week mindfulness‐based cognitive‐behavioral intervention [12]. All barriers reported in this study are modifiable; hence, integrating the TelePDPP into maternal care systems is potentially feasible. Importantly, both patients and clinical staff were reported to appreciate the TelePDPP, primarily due to easy access. Rural patients in particular were reported to value the TelePDPP because its remote‐access delivery addresses their concerns about privacy and stigma, congruent with previous studies [20, 21].

Nurse managers are critical for the integration of the TelePDPP into routine maternal care, as they are primarily responsible for creating, controlling, and organizing the schedule of nurses, and for managing clinical workflow [22]. Clinical staff workflow, identified in both the “barriers” and “recommendations” themes, is a modifiable structural barrier of the TelePDPP. The workflow design of clinical staff is an essential component of integrating the TelePDPP into routine maternal care, in agreement with previous reports [23, 24]. Referring patients to the TelePDPP is not part of the essential duties of clinical staff sampled, which may explain their low knowledge/experience with the TelePDPP, especially those not working in maternal mental health–related units. Corroborating this finding, a study conducted in Canada reported that nurses had lower intention to use and actual use of mobile health applications when the use of mobile health apps was optional compared to being a required component of their job duties [25]. Clinical staff workflow barriers can be modified following suggestions provided by clinical staff. For example, linking the OPEP to the existing patient information portal was stated as a valuable way to facilitate the incorporation of the TelePDPP into clinical staff workflow. For urban clinical staff, this would be linking the OPEP to MyChart, and for rural clinical staff, this would be referring patients from WIC clinics to the OPEP. Rural patients are often reported to have lower socioeconomic status that meets WIC eligibility compared to urban patients [26, 27]; this may be why WIC is a great avenue to refer or provide education about the TelePDPP. Conversely, urban patients have access to MyChart, whereas rural patients visiting the public health clinics do not [28]. Moreover, both groups of clinical staff agreed that downloading the OPEP app for patients in the clinical setting and following up to ensure that they are using the app at subsequent visits will improve flow and encourage patient engagement with the TelePDPP, in line with findings from a previous study [29]. Nurse managers can aid the smooth implementation of these suggestions through their influence as leaders in the clinical settings [30].

Findings from this study revealed that telehealth adoption is context and infrastructure‐dependent because urban clinical staff reported having experience with the OPEP and referring patients to the TelePDPP more frequently compared to rural staff. Similar findings were reported in earlier studies, citing higher rates of telehealth adoption and internet access in urban hospitals as a plausible reason [31]. Conversely, some studies reported more frequent telehealth use among rural medical providers and patients compared to their urban counterparts [32, 33]. Although both rural and urban patients were reported to have experienced some difficulty accessing the OPEP, these barriers were more pronounced among rural patients as identified in previous studies [6, 7, 31]. Stigma was the most commonly reported barrier, congruent with findings from previous studies [34, 35]. Feasible interventions were recommended by rural clinical staff that may decrease this barrier—promoting and marketing the privacy of the TelePDPP and increasing awareness of PD through the TelePDPP. Importantly, as the TelePDPP was delivered via a mobile health app, we expected technology‐related concerns [31]. Indeed, next to stigma, low technology literacy and lack of access to an internet‐enabled phone was another common barrier for rural patients in line with earlier reports [7, 20]. It is crucial to address these barriers to reduce rural‐urban inequity in access to the TelePDPP and other virtual care interventions that may promote health and well‐being among women.

Importantly, the endorsement and promotion of the TelePDPP by medical providers is another important measure that may potentially increase its integration into healthcare systems. Evidence shows that patients are more likely to participate in research when recommended by their medical providers [36]. Other suggestions to promote the uptake of the TelePDPP were summed in the “recommendations” theme, and these are consistent with findings from previous studies [10, 37]. Rural clinical staff opined that community endorsement is core toward promoting the acceptability of the TelePDPP by rural patients; hence, researchers, clinical staff, and hospital administrators may consider promoting the TelePDPP via community engagement in this population. Mollard and colleagues (2016) found that rural women rely on informal networks for the validation of PD treatment and suggested including family, friends, and significant others in the TelePDPP [38].

### 4.1. Implications

Findings from this study may have implications for clinical practice, research, and policy. As clinical leaders, nurse managers are integral to implementing and sustaining telehealth; they can address the barrier of heavy workflow to facilitate integrating the TelePDPP into routine maternal care [39]. Integration of perinatal telehealth programs into rural systems can help address a number of barriers including travel and stigma. Additionally, perinatal telehealth programs allow urban providers to serve rural patients, enhancing access to care. Nurse managers are also involved in health policy making [40]. Hospital policies to promote effective communication between clinical staff and patients (e.g., supporting interpretation in patients’ preferred languages) are warranted to increase patients’ health literacy. The study’s potential implications for technology include improving the user interface of health apps to promote a positive attitude towards its utilization among patients. Furthermore, we suggest that these resources should be at no, or low cost to patients and hospital administrators should have resources in place to support patients with low technology literacy in downloading and logging into apps and utilizing other telehealth technologies as well as assisting with technical difficulties that arise. Finally, future research should evaluate the effectiveness of structured workflow integration strategies and quantitatively assess rural–urban differences in telehealth engagement to inform scalable implementation models.

### 4.2. Strengths and Limitations

To our knowledge, this is the first study to deliver a PD prevention program for both rural and urban patients in Utah using an existing OPEP—and to examine the feasibility of its implementation in healthcare systems. In addition, interviewing both rural and urban clinical staff enhanced the collection of rich data about the facilitators, barriers, and recommendations to promote the uptake of the TelePDPP. Nonetheless, the inadequate experience of rural clinical staff with the TelePDPP and OPEP is a limitation. The focus of the study in a single state in the United States further limits the applicability of findings to women and clinicians in other states as rural and urban characteristics may vary across settings. In addition, findings may not be applicable in settings with low prevalence of PD, settings where there is no stigma regarding PD or settings with low technology literacy or use. We used a qualitative design, which is prone to researcher bias and makes anonymity difficult [41]; nonetheless, we applied several methods to maintain rigor including maintaining reflexivity and trustworthiness through team‐based coding, audit trail of transcripts, reflexive and analytic memos, and detailed contextual descriptions and representative quotes.

## 5. Conclusions

The TelePDPP was perceived as accessible and valuable; however, structural barriers—including workflow constraints, provider engagement variability, and digital access inequities—limited optimal integration. Rural–urban differences in infrastructure, stigma, and referral processes highlight the need for context‐sensitive implementation strategies. Embedding telehealth interventions within formal clinical workflows, strengthening provider endorsement, and investing in digital inclusion are essential to advancing equitable access to perinatal mental health support. Technology companies investing in social media apps should prioritize maintaining the privacy and confidentiality of their clients to promote a positive experience for women who may choose to access mental health interventions via closed, private social media groups. Additionally, the provision of vetted, evidence‐based social media groups is needed to combat online misinformation.

## Funding

This work was supported by the National Institutes of Health, National Institute of Nursing Research (Grant No. RF1NR020841 [PI: Latendresse, G]).

## Disclosure

The sponsor had no role in the study design; in the collection, analysis and interpretation of data; in the writing of the report; and in the decision to submit the article for publication. The content is solely the responsibility of the authors and does not necessarily represent the official views of the University of Utah or the National Institutes of Health.

## Conflicts of Interest

The authors declare no conflicts of interest.

## Supporting Information

Additional supporting information can be found online in the Supporting Information section.

## Supporting information


**Supporting Information** Supporting File 1 contains the SRQR checklist used as a guide to conduct and report findings from the study. The focus group guide containing a list of questions asked during focus groups is included in Supporting File 2. Finally, Supporting File 3 contains additional quotes from participants.

## Data Availability

The data that support the findings of this study are available from Gwen Latendresse upon reasonable request.
