# Supplementary material for: Implementing a Telehealth Perinatal Depression Prevention Program: Perspectives of Rural and Urban Clinical Staff
Source: J Nurs Manag. 2026 Oct 9;2026:4109971. doi: 10.1155/jonm/4109971 (PMC13651005; doi:10.1155/jonm/4109971)
Supplement: Supplementary file 1 [file JONM-2026-4109971-s001.zip › 4109971.f3.pdf]

Supplement 3 Additional Quotes from Participants

Themes	*Subthemes*	*Examples of quotes*
Facilitators	Facilitators for Patients (as reported by clinical staff)	*"… I like that it's something that you can do from home and the education… it's something they can do at home on their own…like they can still maintain their privacy, I guess." [Rural Clinical staff, 12/10/24]*<br><br>*"… when I did work with, I don't know if you know Dr. EC. She pushed it a ton. So when I was with her occasionally in MFM, she would push it a lot. And I did get some good feedback, nothing specific from patients with hers, but I know they said it was really helpful, and they liked going to it…" [Urban Clinical staff, 10/25/24]*
	Facilitators for Clinical staffs	*"The feedback I've heard from other nursing directors from other health departments is that the women appreciate it. They appreciate the resource…" [Rural Clinical staff, 6/20/24]*<br><br>*"…we actually have a Spanish clerk, which has been really helpful for doing these things." [Rural Clinical staff, 12/11/24]*
Barriers	Barriers for Patients (as reported by clinical staff)	*"… maybe like a cultural kind of, I'm not sure, but I think maybe some cultures are less inclined to seek mental health help… maybe it's more of a stigma for them to seek it out." [Urban Clinical staff, 10/23/24]*<br><br>*"From what I've heard, I feel like just being in a frontier county, everybody knows everybody. So, there is that stigma of going to see a therapist or getting mental health help. You feel like, okay, well everybody's going to know what I'm doing…" [Rural Clinical staff, 12/10/24]*<br><br>*"A problem that we do have at the R Clinic is the language barrier with the patients. We do have a lot of patients that speak different languages. So, we haven't been really using the [OPEP] with them just because of that barrier..." [Urban Clinical staff, 3/13/24]*<br><br>*"I think not having access to a device would definitely hinder them being able to use it, especially if they like don't have minutes all the time. They have one of*

		those type of phones where they buy minutes…" [Urban Clinical staff, 3/13/24]<br><br>*"… I've had a couple that said they couldn't find the postpartum screening on the app." [Rural Clinical staff, 12/11/24]*<br><br>*"… sometimes there's a lot of information that we give to the patient…" [Urban Clinical staff, 3/13/24]*
	Barriers for Clinical staffs	*"…sometimes even the culture within our own health department is trying to figure out how this could be easy for us to promote to patients. How can get embedded into the WIC (Special Supplemental Nutrition Program for Women, Infants, and Children) screening. A lot of times we hear that there's not enough time. So just trying to figure out, even for our own staff that are already trying to do so many things in one visit, how to promote it to them in the ease of use." [Rural Clinical staff, 12/11/24]*<br><br>*"If we had a patient with mental health issues that was pregnant or postpartum, we have a provider, Dr. [G], that was working with postpartum mental wellness. That was our go-to. I don't even think postpartum I would say, "Hey, go to the [OPEP] and look at it…" [Urban Clinical staff, 10/25/24]*<br><br>*"I did not know about the mental health part. I mean I use a lot of the breastfeeding videos and a lot of the information there when moms are calling me prenatally to give them some education base..." [Urban Clinical staff, 10/25/24]*<br><br>*"Okay, let's pull the partner and the child away and let the mom just sit and think for a minute and take this questionnaire." [Rural Clinical staff, 12/10/24]*<br><br>*"One thing I noticed and I'm not sure if this was just me not knowing how to use the app properly or if this was a problem with patients too, I thought it was hard to navigate because I had a hard time like finding specific topics in there, even with the search function, because it seemed like the results came up with a lot of like articles that I had to dig through to find specific information." [Urban Clinical staff, 3/13/24]*

Recommendations	Endorsements	*"…if there's any updated flyer or even a social media post that we could share on our Facebook and Instagram pages, I know we've really been working on getting a lot more traction on those pages and that might help as well." [Rural Clinical staff, 12/10/24]*
	Education	–
	Marketing and promotion	*"I think that having even just, either hotlinks or something that could be placed into a MyChart message would make it like these are the videos I want you to watch instead of saying to them, "Okay, pull up your [OPEP] app. You're going to go under this tile. You're going to" that kind of thing because they're so sleep deprived, and they can't even follow my instructions." [Urban Clinical staff, 10/25/24]*<br><br>*"… for me, I worry that maybe the patient will think I'm categorizing them as "You look like you need mental health help" which is a bad way to look at it because, like Amy said, everyone could use it and it creeps up on you, so it's good to know. So, maybe for me, something helpful would be saying like, "Just so you know, we tell this to all our patients. If you need mental health help, this is where you can get some." [Urban Clinical staff, 10/23/24]*
	Workflow enhancement	*"What I was thinking was maybe we could help the patients download the app and then switch their settings to whatever language they speak" [Urban Clinical staff, 3/13/24]*<br><br>*"… getting an in-person interpreter that we have and make the questions make sense instead of a direct translation, that might be a little bit more helpful with those patients and making sure that we are screening them as best as we can." [Urban Clinical staff, 3/13/24]*<br><br>*"I do think that would be kind of helpful, to have that best practice alert, even if it could come up like after they've filled out those scales on their intake stuff and maybe send them some information before their visit even. So, that way if they have questions about it, they can ask us when they're in for their appointment, give them like a little time to mentally prepare and have the idea in mind." [Urban Clinical staff, 3/13/24]*

		"Would there be a way to like as a practitioner see what our patients are accessing? Like I'd be like, "Oh, I saw you watched this video. What questions did you have about that? Or like, oh this is a mom that's clicked on like the mental health tab like 50 times. I should maybe check in with her and see if she's got the resource she's needing or like what is it in that area that's helping her?..." [Urban Clinical staff, 10/25/24]
	Intervention delivery	*"… Is anything that they post in that group for support going to be private as far as like the rest of their Facebook profile goes? Just wondering if maybe someone would be a little inhibited if they don't want all of their Facebook friends to see them posting about their postpartum depression. Is it going to be like just limited to that group?" [Urban Clinical staff, 3/13/24]*
