# Supplementary material for: Implementing a Telehealth Perinatal Depression Prevention Program: Perspectives of Rural and Urban Clinical Staff
Source: J Nurs Manag. 2026 Oct 9;2026:4109971. doi: 10.1155/jonm/4109971 (PMC13651005; doi:10.1155/jonm/4109971)
Supplement: Supplementary file 1 [file JONM-2026-4109971-s001.zip › 4109971.f2.pdf]

Supplement 2 Focus Group Guide

Initial Questions:

1. Who attended the last focus group in June? Are you currently using YoMingo?

2. Are you using YoMingo specifically to refer rural patients for mental wellness education during pregnancy and postpartum?

   a. What is preventing you from using it?

   b. For those who are using it, what is facilitating the process?

YoMingo Feedback:

3. What is working well with the YoMingo patient education portal for educating rural patients about mental wellness during pregnancy?

   a. If you attended the focus group in June, have you had more exposure to YoMingo since then?

4. What is not working well with the YoMingo patient education portal for educating rural patients about mental wellness during pregnancy?

5. What specific barriers prevent you from referring rural patients to mental wellness education during pregnancy and postpartum?

6. What are your suggestions for improving the process of referring rural patients for mental wellness education using YoMingo?

7. What do you think about using YoMingo for delivering the PD prevention program?

8. Tell us about your experiences with referring rural patients to YoMingo and/or the PD prevention program.

If No One Is Using YoMingo:

1. What would help you in the process of referring every rural patient to YoMingo?

   a. For rural patients with mental health issues, what would help in referring them to the mental wellness education and the PD prevention program?

2. What are the barriers to referring every rural patient to YoMingo?

   a. For rural patients with mental health issues, what are the barriers to referring them to the mental wellness education and the PD prevention program?

3. Do you have any suggestions to help with this process?
